# The chronergy of recombinant streptokinase thrombolysis in acute myocardial infarction

**DOI:** 10.3892/etm.2013.980

**Published:** 2013-02-27

**Authors:** ZHONG-MING WANG, YA-BING LIU, QI-CHEN JIN, XUE-QI WANG, MENG DAI, HUI SHAO, WEN-PING ZHAO, QIU-LI DONG, SHU-PING WANG, HAI-TAO ZHANG, LI-CHA KONG, SHAO-YUN LIU, DONG-YING WANG

**Affiliations:** 1 Second Department of Cardiology, General Hospital of North China Petroleum Administration;; 2 Department of Internal Medicine, Jingxia Hospital of North China Petroleum Administration, Renqiu, Hebei 062552;; 3 Xiangya School of Medicine, Central South University, Changsha, Hunan 410013;; 4 Department of Science and Technology, Jingxia Hospital of North China Petroleum Administration, Renqiu, Hebei 062552;; 5 Department of Cardiology, Affiliated Hospital of Hebei University, Baoding, Hebei 071000;; 6 Department of Radiology, Jingxia Hospital of North China Petroleum Administration, Renqiu, Hebei 062552, P.R. China

**Keywords:** acute myocardial infarction, chronergy of onset, thrombolytic therapy, morning resistance to thrombolysis

## Abstract

The aim of this study was to explore the chronergy of intravenous recombinant streptokinase (r-SK) in patients with acute myocardial infarction (AMI). A total of 114 patients were divided into two groups according to the time of AMI onset: the morning onset (6:01–12:00, n=53) and non-morning onset (12:01–06:00, n=61) groups. The recanalization rate was recorded, as well as anticoagulant and fibrinolytic indices. Statistical analysis was performed to evaluate the recanalization rate following thrombolysis, as well as the anticoagulant and fibrinolytic activities. The recanalization rates following thrombolysis in the morning onset and non-morning onset groups were 60.4 and 82.0%, respectively (P<0.05). The level of plasminogen activator inhibitor-1 (PAI-1) antigen was significantly higher in the morning onset group compared with that in the non-morning onset group (P<0.05). This indicated a resistance to r-SK thrombolysis in the morning at the early stage of AMI, which possibly correlates with increased PAI-1 antigen levels and activity.

## Introduction

Through more than ten years of extensive studies concerning reperfusion therapy, particularly through stage three clinical trials of thrombolytic therapy for myocardial infarction, it has been identified that coronary artery recanalization plays a crucial role in the prognosis of patients with acute myocardial infarction (AMI). It is well known that AMI onset presents characteristic circadian variations involving a definite morning peak between 6:00 and 12:00 a.m., particularly between 9:00 and 10:00 a.m. (1). One important factor accounting for the high incidence of AMI onset in the morning is the rapid increase in physical and/or mental activities, as well as in blood pressure, in the first few hours after awakening (2–4).

Percutaneous coronary intervention (PCI) and systemic intravenous thrombolytic therapy have been recognized as effective methods for clearing the affected blood vessel. However, the benefit of PCI is less than that of thrombolysis with regard to mortality reduction when the PCI-related time is longer than 62 min (5,6). If the ‘prime time’ of reperfusion is missed, the survival rate drops and recanalizing the affected blood vessel as soon as possible allows for a 25% reduction in the fatality rate (7). As PCI is not available for patients with AMI in a number of hospitals in China, the recanalization rate of systemic intravenous thrombolytic therapy in the treatment of coronary artery disease is extremely low. Along with the comprehensive development of thrombolytic efficacy for AMI, increasing attention has been drawn to a factor that affects thrombolytic efficacy: resistance to thrombolysis in the morning (8–10). Our previous study focused on the chronergy of thrombolytic therapy for AMI, which to date, has been limited to recombinant tissue plasminogen activator (rt-PA) and urokinase (UK). It was identified that the recanalization rate of thrombolysis with streptokinase (SK) is high. However, SK has been utilized as a drug to treat myocardial infarction only since the 1960s (11). Currently, the main SK thrombolytic drugs officially approved worldwide for clinical use include SK and recombinant SK (r-SK). Whether resistance to r-SK intravenous thrombolysis exists in the morning has not yet been reported.

The current study aims to explore whether morning resistance exists in r-SK intravenous thrombolysis in the treatment of AMI and to investigate the related anticoagulant and fibrinolytic indices.

## Materials and methods

### Subjects

A total of 114 study subjects with AMI were selected from patients at the General Hospital of North China Petroleum Administration affiliated to Hebei Medical University between July 2008 and December 2010. This study was conducted in accordance with the Declaration of Helsinki and with approval from the Ethics Committee of the General Hospital of North China Petroleum Administration affiliated to Hebei Medical University. Written informed consent was obtained from all participants. These subjects accorded with the following conditions: i) diagnostic standard of AMI; ii) thrombolytic therapy within 4 h of AMI onset; iii) exclusion of chronic myocardial infarction, taking aspirin or β-receptor blockers prior to onset, serious lung infection and serious liver disease. The selected patients were divided into two groups according to the time of AMI onset: the morning onset group (6:01–12:00, n=53) with 35 males and 18 females aged 39–80 years, and the non-morning onset group (12:01–06:00, n=61) with 42 males and 19 females aged 36–79 years. There were no statistical differences in gender, history of high blood pressure, history of diabetes or infarction position and heart function (P>0.05).

### Recording methods

Symptom onset, duration and start time of thrombolysis in patients were recorded. The thrombolysis was performed according to ‘References of acute myocardial infarction intravenous thrombolytic therapy’ edited by the editorial committee of Cardiovascular Disease magazine in 1996. Briefly, 300 mg aspirin was chewed immediately and 20,000 U/kg 0.9% sodium chloride solution with r-SK solution (Qingdao Guoda Biopharmaceutical Co., Ltd., Qingdao, China) was intravenously administered to the patients within 60 min. An 18-lead electrocardiogram (ECG) was utilized to continuously monitor ECG parameters at 0, 30, 60, 90, 120 min after thrombolysis. Myocardial enzymes and troponin T were measured 6, 12, 14, 16, 24 h after the onset. Two blood samples (each 1.8 ml) were collected from the antecubital vein without tourniquet at 30 and 120 min after thrombolysis, into anticoagulant tubes containing 0.38% citric acid. The blood samples were immediately centrifuged at 3,000 rpm for 20 min and the plasma was stored at −70°C for further detection. Other pharmaceuticals, including nitric acid ester, statins, β-receptor blockers and angiotensin-converting enzyme (ACE) inhibitors were administered to patients with acute myocardial infarction. The standard of coronary artery recanalization (indirect indications 120 min after thrombolysis) was as described in a previous study (12). The anticoagulant activity was evaluated by measuring the antithrombin III (AT III) activity. Fibrinolytic activity was assessed using a dynamic method, that is, by measuring PAI-1 antigen and α2-antiplasmin (α2-APL) antigen levels. Additionally, the PAI-1 dissolubility was detected using a double antibody sandwich enzyme-linked immunosorbent assay (ELISA). Levels of D-dimer (D-D) were quantified using a double antibody sandwich ELISA with monoclonal antibodies.

### Statistical analysis

Data were analyzed with SPSS 10.0 software (SPSS Inc., Chicago, IL, USA) and were presented as mean ± standard deviation. The variation of means was assessed by analysis of variance or F-test. Chi-square test was performed using cross-tabulation (RxC) multiple group comparison to analyze the recanalization rate.

## Results

### General information

The recanalization rates following thrombolysis in the morning onset and non-morning onset groups were 60.4 (32/53 cases) and 82.0% (50/61 cases), respectively, which were significantly different (P<0.05).

### Anticoagulant and fibrinolytic activities prior to thrombolysis

The comparison of anticoagulant and fibrinolytic activities in the two groups prior to thrombolysis is summarized in Table I.

### Anticoagulant and fibrinolytic activities following thrombolysis

The comparison of anticoagulant and fibrinolytic activities in the recanalized and non-recanalized patients before and at 30 and 120 min after thrombolysis is shown in Table II. At these three time-points, the PAI-1 antigen level and PAI-1 activity in non-recanalized patients were significantly higher than in recanalized patients, respectively (P<0.01).

## Discussion

The aim of our study was to observe whether resistance to r-SK thrombolysis exists in the morning; however, the results of this study are contrary to our expectations. The recanalization rate following r-SK intravenous thrombolysis in the morning onset group was significantly lower than that in the non-morning onset group, which indicates that resistance to r-SK thrombolysis for the treatment of AMI exists in the morning. The level of PAI-1 antigen and its activity is significantly higher in non-recanalized patients than in recanalized patients. In addition, the higher level of PAI-1 antigen and its activity in non-recanalized patients are indicated by i) the differences in anticoagulant and fibrinolytic activities prior to thrombolysis between recanalized and non-recanalized patients and ii) the changes in anticoagulant and fibrinolytic activities that occur after thrombolysis. Thrombolytic therapy should be considered instead of PCI when the patients have a long history of AMI (13–15); however, it is difficult to dissolve thrombi in the coronary artery due to the decreased fibrinolytic activity induced by an increased level of PAI-1. Therefore, the recanalization rate of r-SK may be higher than that of UK at times other than the morning.

Our study reveals a relatively high level of PAI-1 in the morning onset group. When the coronary artery is injured, the release of PA is reduced, while physiological and pathological PAI-1 levels are higher in the morning regardless of the health of the patient. During this period the platelet activity enhances and fibrinolytic activity weakens, which facilitates thrombosis of the coronary artery (16–18). Due to the circadian variation of PAI-1, the thrombolytic efficacy is improved in the afternoon compared with that in the morning and the rhythm of thrombolytic resistance is not related to differences in the thrombolytic preparations. The timely and accurate understanding of AMI chronergy helps to confirm thrombolytic therapy and improve the prognosis following PCI (19). There is no significant difference in heart function between direct PCI and PCI following thrombolytic recanalization (20–22). The thrombolytic time was not consistent with the onset time in a number of cases, which may have affected the results to a certain extent. The optimal solution for AMI patients with a morning onset is to perform PCI or transfer the patients to a tertiary hospital where PCI may be conducted directly.

## Figures and Tables

**Table I:** Comparison of anticoagulant fibrinolytic activity in the two groups prior to thrombolysis.

Testing index	Morning onset group (n=53)	Non-morning onset group (n=61)
AT-III activity (%)	93.64±12.22	93.89±11.91
PAI-1 antigen (*μ*g/l)	39.73±4.63^a^	37.31±4.61
PAI-1 activity (AU/ml)	17.08±3.59	17.16±3.57
α2-APL antigen (*μ*g/l)	90.28±15.30	90.30±14.72
D-D (mg/l)	0.25±0.13	0.25±0.10

Data are presented as mean ± standard deviation.

a P<0.05, compared with the non-morning onset group. AT-III, antithrombin III; PAI-1, plasminogen activator inhibitor 1; APL, antiplasmin; D-D, D-dimer.

**Table II:** Comparison of anticoagulant fibrinolytic activity in the recanalized and non-recanalized patients before and at 30 and 120 min after thrombolysis.

Testing index	Recanalized patients (n=82)	Non-recanalized patients (n=32)
AT-III activity (%)		
Before thrombolysis	97.18±12.64	98.08±6.17
30 min after thrombolysis	62.65±6.94	63.75±6.42
120 min after thrombolysis	49.18±5.75	50.09±4.34
PAI-1 antigen (*μ*g/l)		
Before thrombolysis	36.09±4.50^a^	42.82±3.32
30 min after thrombolysis	35.28±4.18^a^	42.46±4.63
120 min after thrombolysis	35.32±4.10^a^	42.36±5.18
PAI-1 activity (AU/ml)		
Before thrombolysis	15.25±2.69^a^	17.09±3.69
30 min after thrombolysis	13.29±4.56^a^	16.48±4.50
120 min after thrombolysis	11.80±3.88^a^	16.90±4.22
α2-APL antigen (*μ*g/l)		
Before thrombolysis	89.13±15.68	89.71±15.53
30 min after thrombolysis	51.15±7.14	53.80±5.53
120 min after thrombolysis	42.89±7.07	42.87±6.28
D-D (mg/l)		
Before thrombolysis	0.23±0.11	0.24±0.12
30 min after thrombolysis	1.93±0.66	1.97±0.47
120 min after thrombolysis	3.45±1.40	3.40±0.89

Data are presented as mean ± standard deviation.

a P<0.01, compared with non-recanalized patients. AT-III, antithrombin III; PAI-1, plasminogen activator inhibitor 1; APL, antiplasmin; D-D, D-dimer.

## References

[1] Kono T, Morita H, Nishina T (1996). Circadian variations of onset of acute myocardial infarction and efficacy of thrombolytic therapy. J Am Coll Cardiol.

[2] Parker JD, Testa MA, Jimenez AH (1994). Morning increase in ambulatory ischemia in patients with stable coronary artery disease. Importance of physical activity and increased cardiac demand. Circulation.

[3] Kannel WB (2000). Risk stratification in hypertension: new insights from the Framingham study. Am J Hypertens.

[4] Gierach GL, Johnson BD, Bairey Merz CN (2006). Hypertension, menopause and coronary artery disease risk in the Women’s Ischemia Syndrome Evaluation (WISE) study. J Am Coll Cardiol.

[5] Nallamothu BK, Bates ER (2003). Percutaneous coronary intervention versus fibrinolytic therapy in acute myocardial infarction: is timing (almost) everything?. Am J Cardiol.

[6] Boersma E, Maas AC, Deckers JW, Simoons ML (1996). Early thrombolytic treatment in acute myocardial infarction: reappraisal of the golden hour. Lancet.

[7] Antman EM, Anbe DT, Armstrong PW (2004). ACC/AHA guidelines for the management of patients with ST-elevation myocardial infarction-executive summary. A report of the American College of Cardiology/American Heart Association Task Force on Practice Guidelines (Writing Committee to revise the 1999 guidelines for the management of patients with acute myocardial infarction). J Am Coll Cardiol.

[8] Becker RC, Corrao JM, Baker SP (1998). Circadian variation in thrombolytic response to recombinant tissue-type plasminogen activator in acute myocardial infarction. JAPPL Cardiol.

[9] Braunwald E (1995). Morning resistance to thrombolytic therapy. Circulation.

[10] Kurnik PB (1995). Circadian variation in the efficacy of tissue-type plasminogen activator. Circulation.

[11] Beldarrain A, López-Laccomba JL, Kutyshenko VP, Serrano R, Cortijo M (2001). Multidomain structure of a recombinant streptokinase. A differential scanning calorimetry study. J Protein Chem.

[12] Polack B, Schved JF, Boneu B, Groupe d’Etude sur l’Hémostase et la Thrombose (GEHT) (2001). Preanalytical recommendations of the ‘Groupe d’Etude sur l’Hemostase et la Thrombose’ (GEHT) for venous blood testing in hemostasis laboratories. Haemostasis.

[13] Rosenfeld AG (2001). Women’s risk of decision delay in acute myocardial infarction: implications for research and practice. AACN Clin Issues.

[14] Herlitz J, Wireklintsundström B, Báng A, Berglund A, Svensson L, Blomstrand C (2010). Early identification and delay to treatment in myocardial infarction and stroke: differences and similarities. Scand J Trauma Resusc Emerg Med.

[15] Chen W, Woods SL, Puntillo KA (2005). Gender differences in symptoms associated with acute myocardial infarction: a review of the research. Heart Lung.

[16] Krantz DS, Kop WJ, Gabbay FH (1996). Circadian variation of ambulatory myocardial ischemia. Triggering by daily activities and evidence for an endogenous circadian component. Circulation.

[17] Muller JE, Tofler GH (1991). Circadian variation and cardiovascular disease. N Engl J Med.

[18] Guo YF, Stein PK (2003). Circadian rhythm in the cardiovascular system: chronocardiology. Am Heart J.

[19] Trigo J, Mimoso J, Gago P (2010). Female gender: an independent factor in ST-elevation myocardial infarction. Rev Port Cardiol.

[20] Cantor WJ, Fitehett D, Botgundvaag B (2009). Routine early angioplasty after fibrinolysis for acute myocardial infarction. N Engl J Med.

[21] Yan AT, Yon RT, Cantor WJ (2011). Relationship between risk stratification at admission and treatment effects of early invasive management following fibrinolysis: insights from the trial of routine angioplasty and stenting after fibrinolysis to enhance reperfusion in acute myocardial infarction (TRANSFER-AMI). Eur Heart J.

[22] Armstrong PW, WEST Steering Committee (2006). A comparison of pharmacologic therapy with/without timely coronary intervention vs. primary percutaneous intervention early after ST-elevation myocardial infarction: the WEST (Which Early ST-elevation myocardial infarction Therapy) study. Eur Heart J.

